# Prognostic Significance of ADAM17 for Gastric Cancer Survival: A Meta-Analysis

**DOI:** 10.3390/medicina56070322

**Published:** 2020-06-29

**Authors:** Peng Ni, Mingyang Yu, Rongguang Zhang, Mengya He, Haiyan Wang, Shuaiyin Chen, Guangcai Duan

**Affiliations:** 1 Department of Epidemiology, College of Public Health, Zhengzhou University, Zhengzhou 450001, China; nipeng17@163.com (P.N.); yumingyang2019@126.com (M.Y.); hemengyazz@163.com (M.H.); why193117@163.com (H.W.); sychen@zzu.edu.cn (S.C.); gcduan@zzu.edu.cn (G.D.); 2 College of Public Health, Hainan Medical University, Haikou 571199, China

**Keywords:** ADAM17, gastric cancer, prognosis, meta-analysis

## Abstract

*Background and objectives*: The prognostic role of a disintegrin and metalloproteinase (ADAM) 17 has been widely assessed in gastric cancer. However, the results are inconsistent. We performed a meta-analysis to evaluate the prognostic significance of ADAM17 and its association with clinicopathological parameters. *Methods:* The databases of PubMed, Web of Science, and Embase were searched for relevant articles published up to April 2020. The reported hazard ratios (HRs) and odds ratios (ORs) and their corresponding 95% confidence intervals (CIs) were pooled to evaluate the strength of the association. Stata 12.1 was used to perform statistical analyses. *Results:* Seven studies, including 1757 patients, were screened for the meta-analysis. Compared with the high ADAM17 expression group, the pooled HR was higher in the low ADAM17 expression group (HR = 2.04, 95% CI 1.66–2.50; *I*^2^ = 18.1%; *p* = 0.299). High ADAM17 expression was also related to the tumor node metastasis (TNM) stages (OR = 4.09, 95% CI 1.85–9.04; *I*^2^ = 84.1%; *p* = 0.000), lymph node metastasis (OR = 3.08, 95% CI 1.13–8.36; *I*^2^ = 79.7%; *p* = 0.007), and ages (OR = 1.65, 95% CI 1.24–2.21; *I*^2^ = 0%; *p* = 0.692) of the gastric patients. *Conclusions*: This meta-analysis revealed that ADAM17 is a significant biomarker for poor prognosis in gastric cancer.

## 1. Introduction

Gastric cancer (GC) is one of the most common malignancies and the second leading cause of cancer-related death [1]. There were 9.6 million cancer deaths worldwide in 2018, and 8.2% of them were caused by GC [1]. As a result, stomach cancer has become a major public health issue. The disease not only imposes an economic burden, but also affects quality of life for stomach cancer patients [2]. Gastric cancer is a multifactorial disease, and many factors can increase the risk of stomach cancer, such as Helicobacter pylori infection, family history, alcohol consumption, and smoking [3]. GC cases usually reach an advanced stage before diagnosis and most probably have poor prognosis since the early symptoms of stomach cancer were not obvious [4,5]. Therefore, determining an effective indicator to improve the prognosis is of great significance for improving the quality of life and reducing the mortality rates of GC patients.

The ADAMs (a disintegrin and metalloproteinases) are a multifunctional family of proteins, many of which are associated with the formation and progression of cancer [6,7]. Several studies showed that adamalysines (ADAM proteins) were highly expressed in gastric cancer and played an important role in gastric cancer proliferation and invasion [8,9,10]. Adamalysines might be involved in the pathogenesis of gastric cancer and involved in EGFR signaling pathway and TGF-β/Smad pathway [11,12]. ADAM17, also named the tumor necrosis factor-alpha converting enzyme (TACE), is a member of the ADAM protein family, which is associated with inflammation and cancer [13,14]. Recently, studies reported the relationship between ADAM17 and certain cancers, including the esophageal squamous cell carcinoma, prostate cancer, breast cancer, colorectal cancer, and stomach cancer [12,15,16,17,18,19,20,21]. In many tumors, high expression of ADAM17 was associated with poor prognosis [19,22,23]. The overexpression of ADAM17 may enhance the ability of migration of GC cells and tumor growth [19,22,24]. ADAM17 may be an important biomarker for prognosis of gastric cancer patients and play an important role in the development and progression of gastric cancer [25]. In addition, inhibition of ADAM17 can reduce the invasion of tumor cells induced by hypoxia [26].

The association between ADAM17 and gastric cancer has been described in several studies, but the significance of ADAM17 for the prognosis of the cases is inconsistent among reports [22,24,25,27,28]. Hence, we performed this meta-analysis to assess the prognostic value of ADAM17 and evaluate the relationship between ADAM17 expression and clinicopathological parameters in GC.

## 2. Materials and Methods

### 2.1. Literature Search Strategy

Literature was searched in PubMed, Embase, and Web of Science until April 2020. The main search keywords are as follows: “stomach tumor” or “stomach cancer” or “stomach carcinoma” or “gastric tumor” or “gastric cancer” or “gastric carcinoma” and “ADAM17 protein” or “TNF-alpha converting enzyme” or “TNF alpha converting enzyme” or “ADAM-17” or “Tumor necrosis factor-alpha converting enzyme” or “Tumor necrosis factor alpha converting enzyme” or “TNF-alpha convertase” or “ADAM-17 protein” or “ADAM17 protein”. In addition, references in original studies and reviews were manually searched.

### 2.2. Study Selection

Studies were included if they met the following criteria: (1) the participants were patients with GC; (2) they reported ADAM17 level and hazard ratios (HRs) with 95% confidence intervals (CIs) of patients or provided the ADAM17 levels and clinical information; (3) ADAM17 had an explicit inspection method (e.g., IHC, ELISA, rt-PCR, etc.). Studies would be excluded if (1) the studies involved the same population that the studies have incorporated; (2) the articles were case reports, letters, comments, reviews, or conference summaries.

### 2.3. Data Extraction and Quality Assessment

The two authors (P.N. and M.Y.) independently extracted data. It included age, gender, the first author of the study, follow-up time, sample size, publication year, HRs, 95% CIs for overall survival (OS). If there was the same cohort of patients in several studies, the article with sufficient information, long follow-up time, and large sample size was selected. 

The two investigators (P.N. and M.Y.) independently assessed the quality of the selected literature using the Newcastle-Ottawa-Scale (NOS) [29]. The score of the quality assessment was from 0 to 9. 

### 2.4. Statistical Analyses

The HRs and corresponding 95% CIs were used to estimate the effect of ADAM17 expression on survival rates. The HRs and 95% CIs would be directly extracted, if they were reported in the original studies. Otherwise, the HRs were estimated using the methods described by Parmaretal et al. [30]. The odds ratios (ORs) and their 95% CIs were used to express the correlation between ADAM17 and clinicopathological parameters. 

The *I*^2^ statistics and Cochran *Q* were used to test the heterogeneity [29]. The value of *I*^2^ statistics was 25%, 50%, 75%, considered as low, moderate, and high heterogeneity, respectively. In addition, *p* < 0.10 was considered statistically significant for the *Q* statistic [31]. The random effects model was employed when the *I*^2^ statistics was more than 50% or the *p*-value was less than 0.1 for the *Q* statistic; otherwise, a fixed effects model was utilized. We conducted a sensitivity analysis to evaluate the stability of the results by excluding a single study at one time. The Begg’s test and Egger’s test were used to assess publication bias [32]. We used Stata 12.1 (Stata Corp, College Station, TX, USA) to perform the analytical process. 

## 3. Results

### 3.1. Characteristics of Studies

**Figure 1:**
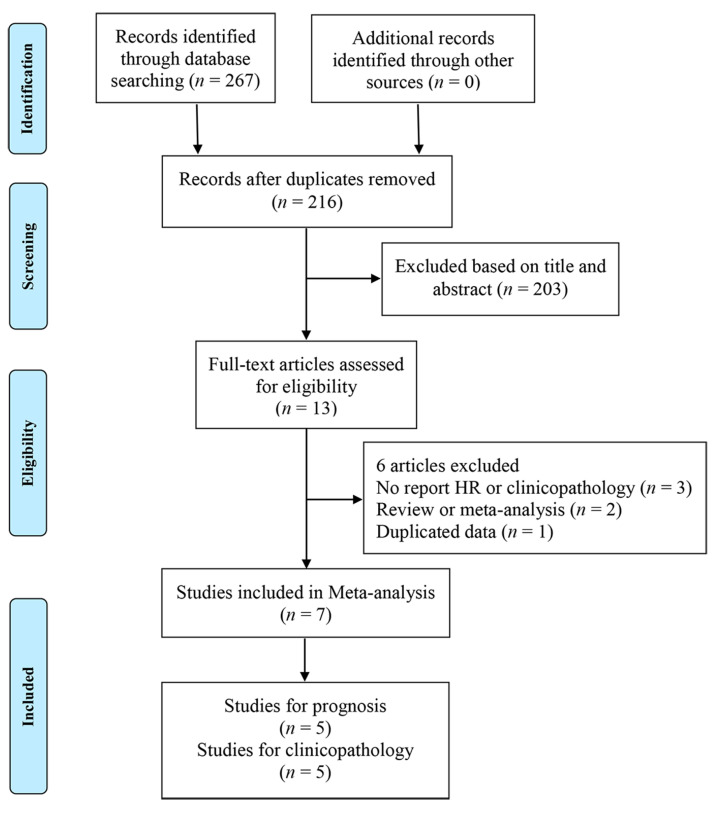
Flowchart of the study selection process. HR: hazard ratio.

We identified 267 potentially relevant articles from PubMed, Web of Science, and Embase databases for the meta-analysis, and 51 of them were excluded because of duplicates. After screening the titles and abstracts, 203 articles were removed, and 13 articles were retrieved for full-text assessment. Finally, seven with a total of 1757 participants were included in this meta-analysis [19,22,24,25,27,28,33]. The selection and exclusion process is presented in Figure 1. Five articles were included for the prognostic value of ADAM17 in GC, and 5 articles were used to explore its association with clinicopathological parameters.

### 3.2. Study Characteristic 

The main characteristics of the eligible studies are in Table 1. Among the seven articles, five were from China [19,22,24,25,27], one from the United States [33], and one from Germany [28]. Seven articles with a total of 1757 patients were published between 2011 and 2019. Sample size ranged from 60 to 486. ADAM17 expression was assessed by immunohistochemistry (IHC). Five studies reported the HR data directly, and five studies mention the clinicopathological parameters. All studies were graded as good quality and all studies adjusted adequately for several potential confounders.

### 3.3. Association between ADAM17 Expression and Overall Survival

**Figure 2:**
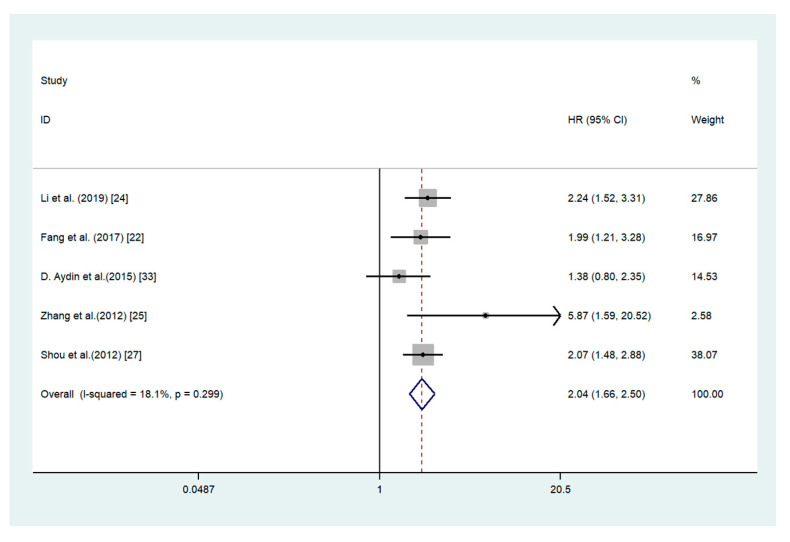
Forest plot for the association between ADAM17 expression and overall survival in gastric cancer. ADAM17: a disintegrin and metalloproteinase 17.

A fixed-effect model was adopted in the study considering the low level of heterogeneity (*I*^2^ = 18.1%; *p* = 0.299). Our study indicated that the poor prognostic effect of ADAM17 in GC was significant. Compared with the high ADAM17 expression group, the pooled HR was higher than in the low ADAM17 expression group (HR = 2.04, 95% CI 1.66–2.50; *I*^2^ = 18.1%; *p* = 0.299) (Figure 2). 

### 3.4. Association of ADAM17 with Clinicopathological Parameters

**Figure 3:**
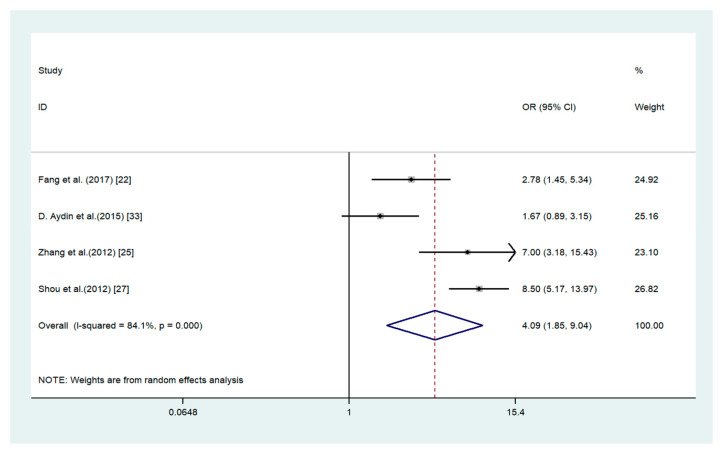
Forest plot for the association between ADAM17 expression and tumor node metastasis in gastric cancer. OR: odds ratio.

**Figure 4:**
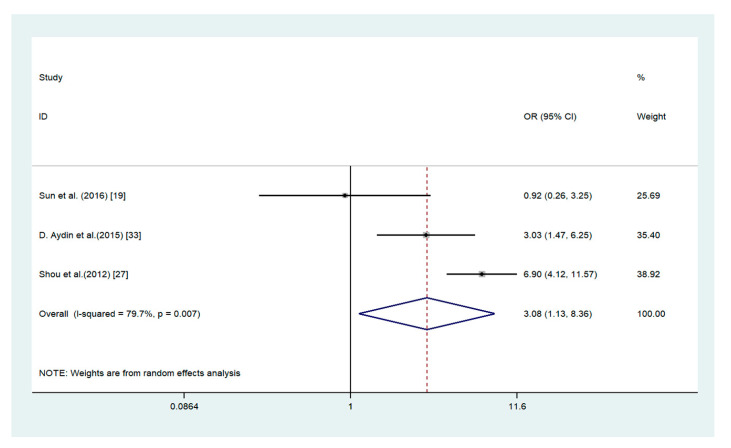
Forest plot for the association between ADAM17 expression and lymph node metastasis in gastric cancer.

**Figure 5:**
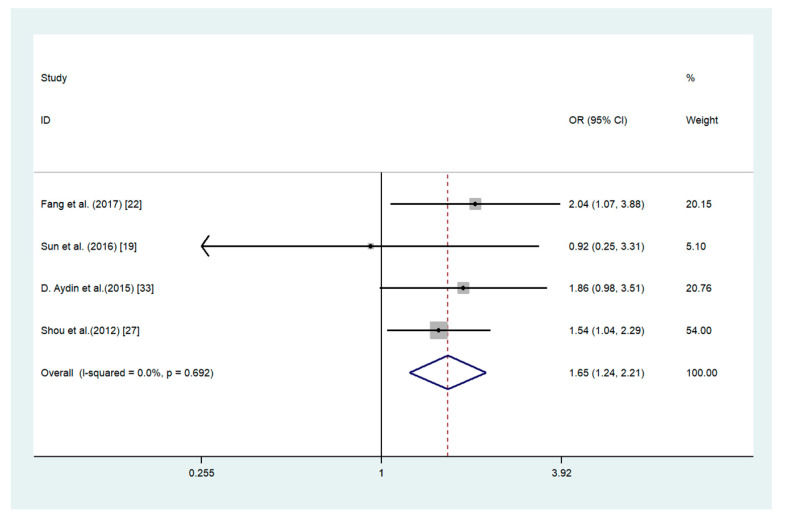
Forest plot for the association between ADAM17 expression and age in gastric cancer.

The elevated expression of ADAM17 was significantly associated with such characteristics as higher TNM stage (OR = 4.09, 95% CI 1.85–9.04; *I*^2^ = 84.1%; *p* = 0.000), lymph node metastasis (OR = 3.08, 95% CI 1.13–8.36; *I*^2^ = 79.7%; *p* = 0.007), and age (OR = 1.65, 95% CI 1.24–2.21; *I*^2^ = 0%; *p* = 0.692) (Figure 3, Figure 4 and Figure 5). There are no association between ADAM17 expression and tumor differentiation (OR = 0.48, 95% CI 0.21–1.11; *I*^2^ = 84.6%; *p* = 0.000) and sex (OR = 0.96, 95% CI 0.75–1.21; *I*^2^ = 23.2%; *p* = 0.260).

### 3.5. Sensitivity Analysis and Publication Bias

Sensitivity analysis showed that all of the estimated pooled HRs corresponding to the omission of each study was inside the 95% CI of the HR estimated from all studies in the overall, indicating that the results of the present study were stable. No significant publication bias was found by the Begg’s test (*p* = 1.000) and the Egger’s test (*p* = 0.493). 

## 4. Discussion

To our knowledge, it is the first meta-analysis discussing the prognostic value of ADAM17 expression in GC patients. As observed, ADAM17 levels were positively associated with poor survival. The risk of pooled probability of mortality was increased by 104% in GC cases, which revealed a worse outcome in GC patients with ADAM17 high expression. The pooled result was reliable according to the results of the sensitivity analysis and no publication bias was detected by Begg’s and Egger’s test. Clinicopathological data analysis showed that ADAM17 level is positively associated with aggressive tumor characteristics.

ADAM17 is related to inflammation and cancers, participates in the tumor proliferation and invasion, and processes more than 80 substrates [13,34]. An increasing number of studies reported that ADAM17 was overexpression in tumors, including the esophageal squamous cell carcinoma, stomach cancer, breast cancer, prostate cancer, colorectal cancer, and others. The ADAM17 was overexpressed in these tumors and associated with the ability of invasion and proliferation. Li et al. reported that ADAM17 activates the Notch and Wnt signaling pathways, which may promote the development of GC [24]. In addition, ADAM17 can promote epithelial mesenchymal transition and proved that it is a therapeutic target for stomach cancer [12].

ADAM17 was correlated with cell proliferation and migration by releasing ligands [35]. A report revealed that the proliferation and migration ability of the cells decreased after inhibiting the expression of ADAM17 and led to the apoptosis of stomach cancer cells [35]. The proliferation and invasion of stomach cancer cells were decreased when the ADAM17 was knockdown from these cells, and the ability of proliferation and invasion was significantly enhanced after these cancer cells were transfected with ADAM17-shRNA [19]. The invasion ability of glioblastoma multiforme (GBM) cells was drastically inhibited when the ADAM17 was knocked down [36]. ADAM17 inhibitor can effectively inhibit the growth and invasiveness of tumor cells, which may be used in cancer treatment in the future [13]. Otherwise, ADAM17 was associated with the prognosis of cancer patients. A report from McGowan et al. indicated that the overall survival was shorter, when comparing the high ADAM17 levels with the low ADAM17 levels for the patients with breast cancer [23]. ADAM17 was associated with GBM and correlated strongly with poor prognosis [36]. 

ADAM17 may be involved in Notch and Wnt signaling pathway, and TGF-β/Smad pathway, which may promote the development of cancer [12,24]. Umemura et al. found that ADAM17 seems to be an important target in the treatment of rheumatoid arthritis [37]. ZLDI-8 (previously named as IAC-8 or inhibitor of ADAM-17 compound No. 8), a novel inhibitor for Notch activating/cleaving enzyme ADAM17, might be a promising therapeutic agent for hepatocellular carcinoma patients [38]. Sun et al. reported that ADAM17 could be a potential target in the treatment of gastric cancer by regulating the EGFR and TNF-α signaling pathways [19]. However, it is not clear whether targeted ADAM17 therapies are effective for cancer treatment and targeted ADAM17 therapies are still in the exploratory stage.

Some studies showed that biological and environmental factors, *helicobacter pylori* infection, excessive alcohol consumption, and smoking, can upregulate ADAM17 expression [39,40,41,42,43]. *Helicobacter pylori* infection may increase the incidence of gastric cancer and can increase the expression of ADAM17 in AGS gastric epithelial cells [44]. In addition, ADAM17 expression level was related to *helicobacter pylori* cytotoxin-associated genes pathogenicity (cagPAI) status and was higher in GC patients with intact cagPAI strains [42].

A previous meta-analysis showed ADAM17 was correlated with TNM stages and distant metastasis, but not correlated with cancer grade of stomach cancer [45]. In our study, we found that ADAM17 was association with TNM stage, lymph node metastasis, and age but not tumor differentiation and sex. In the previous meta-analysis, it only explored the relationship between ADAM17 expression and clinicopathological parameters in stomach cancer but did not assess the prognostic value of ADAM17. In this study, we investigated the relationship between ADAM17 and prognostic value and clinicopathological parameters and found that high expression of ADAM17 was associated with poor prognoses in stomach cancer patients.

The advantages of this meta-analysis are as follows: Firstly, this study performed more comprehensive analysis of the data, including prognostic value and clinicopathological parameters. Secondly, all the data was extracted directly from the articles. However, among the included studies, there were some limitations. Firstly, the definition of level of ADAM17 expression is not consistent in the included studies. Secondly, most of the articles were from China and the results were applicable to Asian populations. Thirdly, our study included few articles, and the results of our study need to be validated by large prospective cohorts.

## 5. Conclusions

Our study shows that lower ADAM17 levels are correlated to the longer OS time in GC and ADAM17 was associated with TNM stage, lymph node metastasis, and age. ADAM17 is a significant biomarker for poor prognosis in gastric cancer.

## Figures and Tables

**Table 1:** Characteristics of the included studies in this meta-analysis.

Study	Year	Country	Male	Sample Size	ADAM17 Source	HR and 95% CI	Study Conclusion	Extraction Type	Analysis Type	Tumor Stage	Detection Method
Li et al. [24]	2019	China	77.70%	193	tissue	2.24 (1.52–3.31)	poor	direct	MV	I–IV	IHC
Fang et al. [22]	2017	China	71.80%	206	tissue	1.99 (1.21–3.28)	poor	direct	MV	I–IV	IHC
Sun et al. [19]	2016	China	35.00%	60	tissue	NA	NA	NA	NA	NA	IHC
D. Aydin et al. [33]	2015	America	64%	156	tissue	1.38 (0.80–2.35)	not	direct	MV	I–III	IHC
Zhang et al. [25]	2012	China	72.73%	220	tissue	5.87 (1.59–20.52)	poor	NA	NA	I–IV	IHC
Shou et al. [27]	2012	China	71.33%	436	tissue	2.07 (1.48–2.88)	poor	direct	MV	I–IV	IHC
Schmuck et al. [28]	2011	Germany	62.55%	486	tissue	NA	NA	NA	NA	NA	IHC

ADAM: a disintegrin and metalloproteinase; HR: hazard ratio; CI: confidence interval; IHC: immunohistochemistry; NA: not applicable; MV: multivariate analysis.
